# Primary cutaneous Leiomyosarcoma on the left iliac region: A rare case report from Syria

**DOI:** 10.1016/j.amsu.2021.102992

**Published:** 2021-10-30

**Authors:** Mohamad Antakle, Mohammed Moutaz Alshaghel, Ghina Ghannam, Mais Al-Ibraheem, Linda Shehade, Sarab Agha, Aladdin Etr

**Affiliations:** a Faculty of Medicine, Aleppo University, Aleppo, Syria; b Department of Pathology, Faculty of Medicine, Aleppo University, Aleppo, Syria; c Department of Plastic Surgery, Faculty of Medicine, Aleppo University, Aleppo, Syria

**Keywords:** Leiomyosarcoma, Cutaneous, Iliac, Soft tissue neoplasms, Surgical procedure

## Abstract

**Introduction:**

and importance: Leiomyosarcoma is a rare aggressive soft-tissue malignancy typically originating from embryonic mesoderm or mesenchymal cell lines in smooth muscles.

Leiomyosarcoma of the skin is termed as “Dermal Leiomyosarcoma”, and is categorized into two subdivisions; superficial cutaneous and deep subcutaneous.

Both types begin either as primary lesions or metastatic lesions from distant sites.

**Case presentation:**

We report the case of a 60-year-old male patient with Primary Cutaneous Leiomyosarcoma (PCL) located in the left iliac region**.**

His history is insignificant and he has no family or genetic history of leiomyosarcoma. The lesion was itchy without any other symptoms and existed 20 years before our evaluation.

A biopsy from the nodule was performed and sent to the pathology department, where the section was stained with smooth muscle actin stain (SMA) and the result was positive.

We referred the patient to a surgeon to excise the nodule. The lesion was excised with a 3cm safety margin, the eradication includes also the of the major iliac muscle.

After one year of follow-up there was no metastasis nor recurrence.

**Conclusion:**

Primary Cutaneous Leiomyosarcoma is a very rare malignancy and it is hard to diagnose without biopsy and pathological examination.

## Highlights

• Primary cutaneous Leiomyosarcoma is a very rare tumor.
• Accurate diagnosis cannot be made without histological and immunohistochemical examination.
• Surgery is the only guaranteed treatment and other methods are still controversial.

## 1 Introduction

Leiomyosarcoma (LMS) is an aggressive soft-tissue malignancy typically originating from embryonic mesoderm or mesenchymal cells in smooth muscles. The term Leiomyosarcoma encompasses a broad spectrum of malignancies with highly variable presentations based on the site of origin, the extremities being the commonest. However, they can remain dormant for a long time [[1], [2], [3], [4]].

Leiomyosarcoma of the skin is termed “Dermal Leiomyosarcoma”, and categorized into two subdivisions: superficial cutaneous and deep subcutaneous [3,5].

Both types begin either as primary lesions or metastatic lesions from distant sites. Primary Cutaneous Leiomyosarcoma (PCL) is considered the rarest subtype with a few reported cases in the literature [3].

Generally, assessing the overall risk of this type of cancer remains difficult due to its rarity. It is highly unpredictable and typically associated with a poor prognosis [6].

We report the case of a 60-year-old male patient with PCL located in the left iliac region. The unusual site of origin of this rare PCL makes this a compelling case report.

This case report has been reported in line with the SCARE Criteria [7].

## 2 Case presentation

**Fig. 1:**
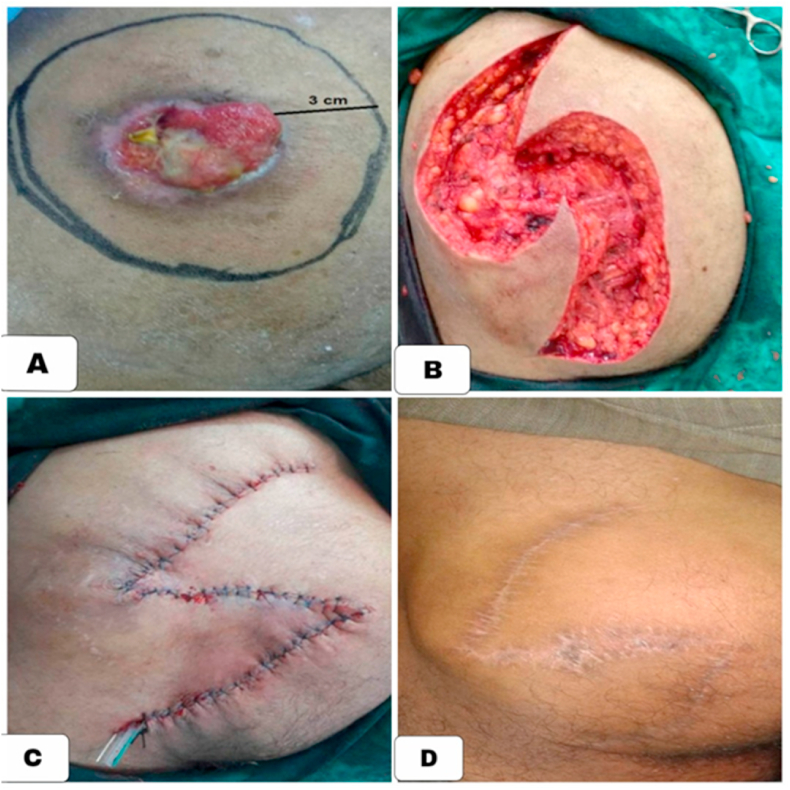
(A) clinical photograph showing the nodule, measuring 3cm diameter with skin ulceration on the left iliac region. (B) During the surgery. (C) The drainage tube. (D) The iliac region after 8 months.

A 60-year-old-male patient presented to the dermatology clinic due to a cutaneous lesion in the left iliac region. His history was insignificant and he had no family or genetic history of leiomyosarcoma. In present history, the lesion was itchy without any other symptoms and existed 20 years before the attendance. In the physical examination, we noted an ulcerative nodule with a 3 cm diameter, moveable with the skin and non-stick with the layer underneath (the muscles) [Fig. 1].

**Fig. 2:**
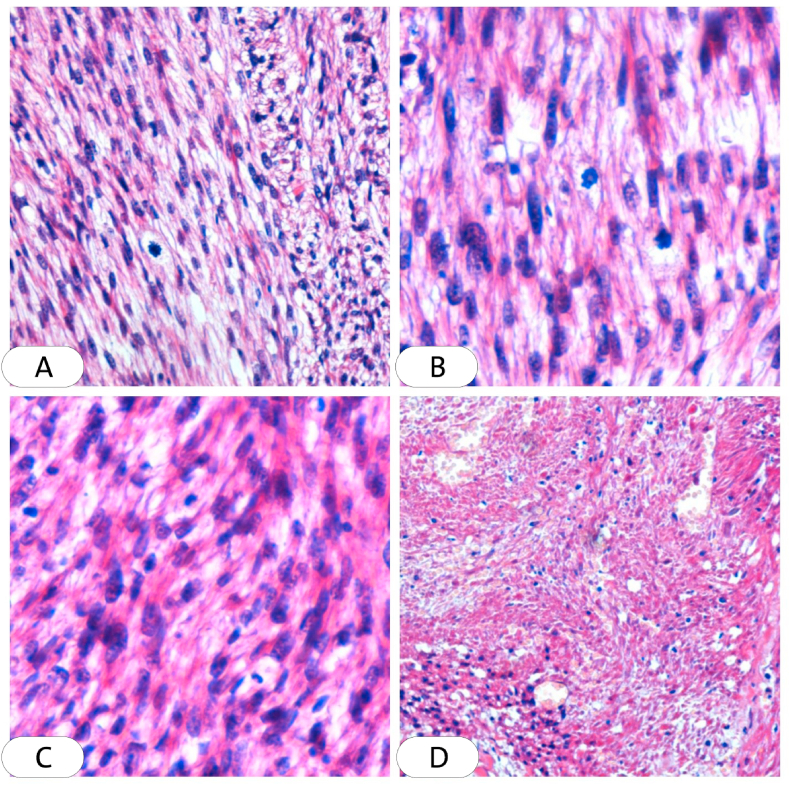
Interlaced fascicles of spindle-shaped cells with elongated (cigar-shaped) nuclei, some fields show atypical mitosis, others show wide areas of haemorrhage and necrosis. (A) Hematoxylin & eosin, X40. (B)(C) Hematoxylin & eosin, X60. (D) Hematoxylin & eosin, X4.

**Fig. 3:**
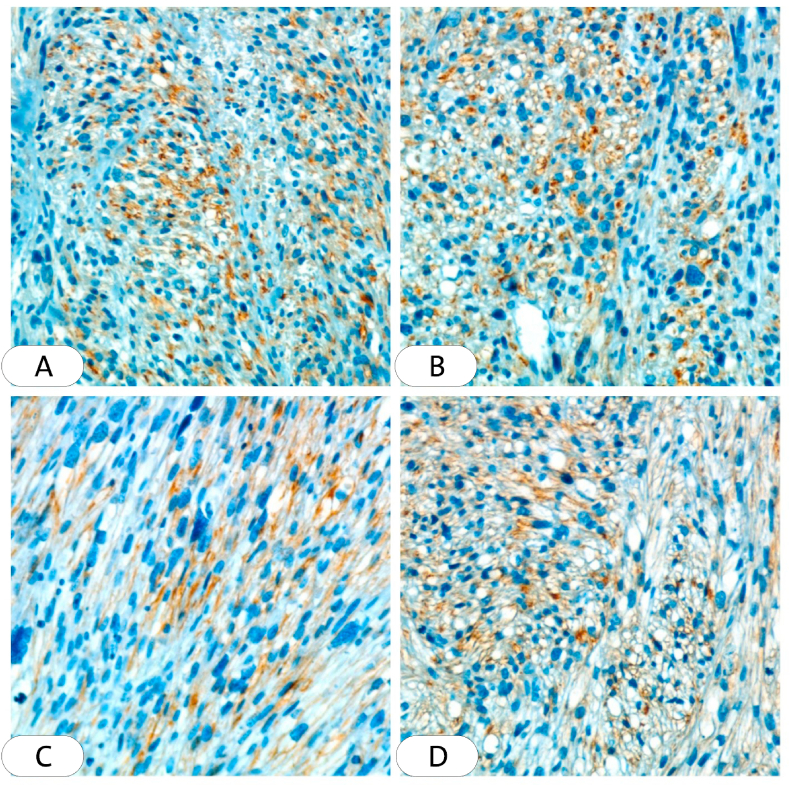
Immunohistochemistry for smooth muscle actin (SMA) (cytoplasmic stain), show diffuse positivity. (A)(B)(D) X10. (C) X40.

**Fig. 4:**
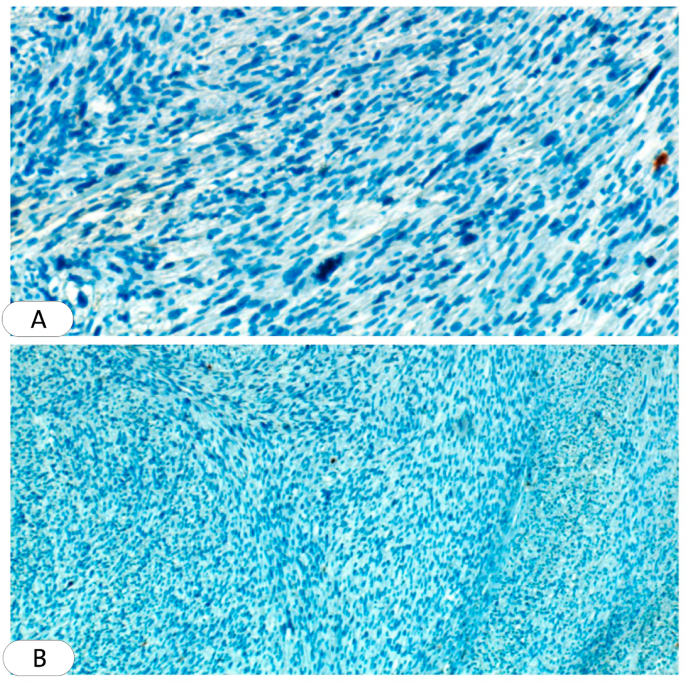
Immunohistochemistry for S100 (nuclear stain), is negative in tumor cells. **(A) X40. (B) X4.**

**Fig. 5:**
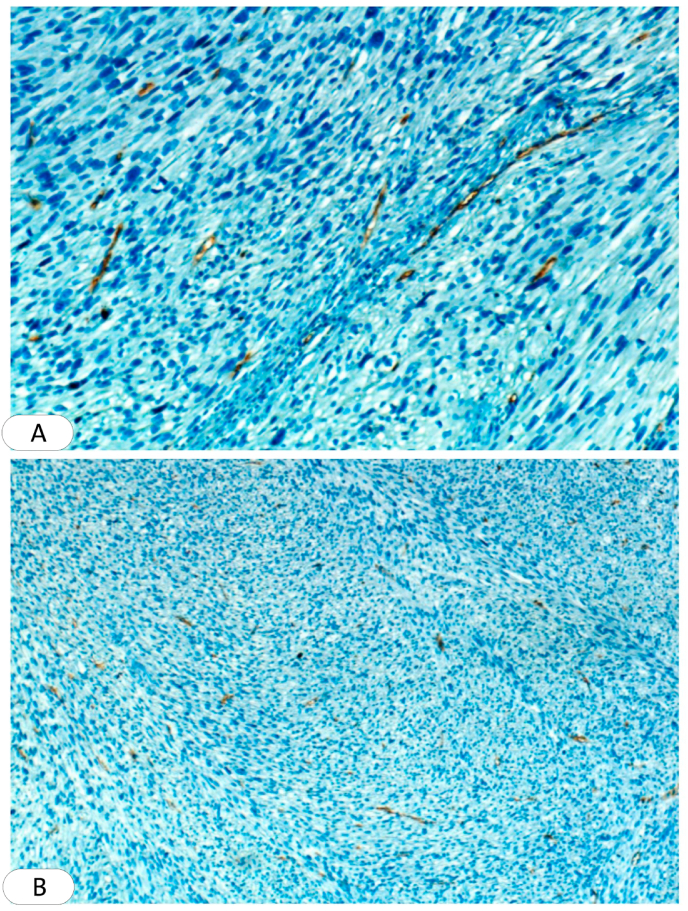
Immunohistochemistry for CD34 is negative in tumor cells. **(A) X40. (B) X4.**

**Fig. 6:**
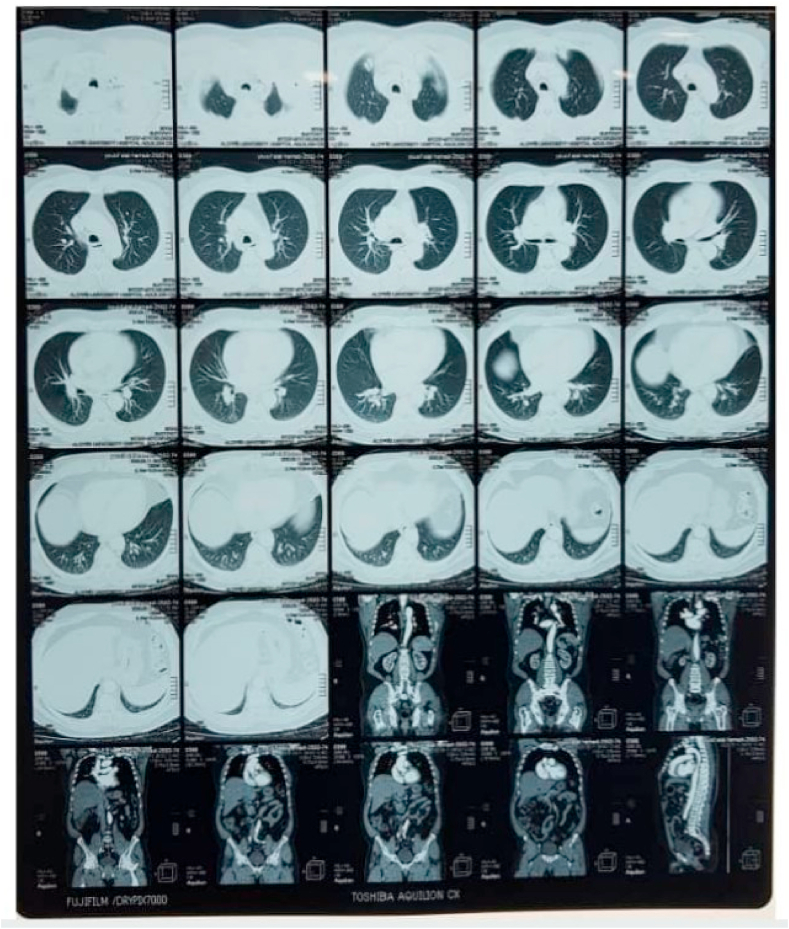
CT with contrast of Chest, abdomen, and pelvis shows normal findings.

Systemic investigations, including complete blood count (CBC), liver and renal function test, and blood sugar level were normal. With these clinical findings, Leishmania and squamous cell carcinoma were considered and a biopsy was required. An excisional biopsy was performed and sent to the pathology department, where one section measuring 3 × 9 × 9 was studied, Hematoxylin and eosin (H&E) sections showed ulcerated epidermis. Interlacing fascicles of elongated spindle-shaped cells with blunt-ended (cigar-shaped) nuclei invading the dermis and parts of subcutaneous adipose tissue. The nuclei revealed variable pleomorphism & hyperchromasia with scattered atypical mitosis. All the deep and lateral surgical borders were free of malignancy [Fig. 2]. The section was also stained with (SMA) which is more sensitive to leiomyosarcoma and the result was positive [Fig. 3]. Negative immunohistochemical staining for S100 and CD34 ruled out malignancies of nervous and vascular origins, respectively [Fig. 4, Fig. 5]. Chest, abdomen, and pelvis CT with contrast were performed to investigate metastasis and they was normal [Fig. 6].

We referred the patient was referred to a senior surgeon to excise the nodule. The pathological examination and the surgical operation have taken place in Aleppo University Hospital. The lesion was eradicated with 3cm safety margins. The excision also included the major iliac muscle. Two localized transposition slides were made, after that, a drainage tube was put, and finally, it was closed by simple sutures [Fig. 1]. The surgery was successful; the patient received the usual postsurgical antibiotics, anti-inflammatories and painkillers. And had been covered with antibiotics, anti-inflammatories, and painkillers after it [Fig. 1]. The follow-up was twice a year in the first year and then once a year until now and no metastatic nor recurrence has been noted so far.

A general physical examination of the patient including the surgical site to monitor any changes after surgery was performed at each follow-up visit.

## 3 Discussion

LMS is an extremely rare malignancy, it composes about 10% of soft tissue tumors that make up 1% of all malignancies [8]. LMS develops on the skin can be classified into superficial or metastases from a distant site with vascular origin such as the uterus or retroperitoneal [9]. Cutaneous LMSs (CLM) are derived from the arrectoress pilorum muscle of the hair follicle in the dermis while subcutaneous emanate from smooth muscle in the walls of arterioles and veins [10,11]. CLM was first described by Montgomery and Winkleman [12,13].

CLM is most common in the lower extremities, but it can appear in any part of the body, rarely in the face [14]. Middle-aged patients (5th – 6th decade) are most likely to be affected by LMS, with male gender predominating (1:3) [14].

The Aetiology of LMS is not yet completely clear, but several factors are believed to have an association such as radiation, trauma, and lupus vulgaris. Malignant transformation of leiomyoma can also be considered as a possible cause [15,16]. The lesion in our case was only itchy without other symptoms, but presenting for 20 years before the attendance raises the possibility that it may be a malignant transformation of leiomyoma.

In a large case series conducted by Fields and Helwig containing 63 LMS skin lesions. The average size was 1.8 cm when presented [16]. The diameter of the lesion in our patient was approximately 3 cm, which is slightly above the recorded median.

Skin changes such as discoloration and ulceration are common symptoms, in contrast, pain is an uncommon symptom but it should be noted that the presence of pain and rapid growth is a sign of a bad prognosis [12,17].

Imaging techniques and clinical manifestations can lead to an initial diagnosis but an accurate diagnosis of LMS can only be made with histological findings and immunohistochemical examination [12]. However, benign findings are not an irrefutable sign of the absence of malignancy [12]. On histopathological examination, LMS consists of well to moderately differentiated spindle-shaped cells. cigar-shaped nuclei and eosinophilic cytoplasm [2]. Immunohistochemically, LMS is positive to SMA, desmin, and h-caldesmon but usually negative to r S-100 protein and cd34 [18].

The best-recommended treatment for LMS is a wide excision of the lesion with appropriate margins of safety (3–5 cm) and a depth that includes the fascia and subcutaneous tissue [19]. We went with this method in our patient, the lesion was removed with wide safety margins, also eradication includes the major iliac muscle and in order to compensate for the cutaneous loss, Z-plasty was applied. Adoption of adjuvant therapy after surgery is still controversial, however, adjuvant therapy is often indicated for high-risk patients or in case of recurrence [19,20]. Some studies adopted the use of Mohs Micrographic Surgery as a treatment for LMS, the result was a decrease in the recurrence of the disease. though, more studies are needed to confirm these results [21].

The prognosis differentiates according to the histological classification in addition to several factors, tumor size more than 5 cm, tumor involves the fascia, high histological grade, high mitotic rate of >20 per 10 high-power fields (HPF), and tumor necrosis of >50% Indicate to bad prognosis [12,15]. As noted above, histological classification plays a role in the prognosis, as cutaneous LMS has significantly lower rates of local recurrence (30–50%) and metastasis (0–10%) compared with subcutaneous LMS that has a recurrence rate of (40–60%) and metastasis of (20–60%) [15].

## 4 Conclusion

As a conclusion, primary cutaneous Leiomyosarcoma is a very rare malignancy and It is hard to diagnose without biopsy and pathological examination.

In our case the patient was 60 year old male with PCL located in the left iliac region with no symptoms besides the itchy feeling in the lesion. Chest, abdomen, and pelvis CT scan with injection were performed to investigate metastasis and there was none. The lesion was eradicated with 3cm safe borders by the surgeon after one year of follow-up there was no metastasis nor recurrence has been noted.
